# Comparative analysis of thermophilic and mesophilic proteins using Protein Energy Networks

**DOI:** 10.1186/1471-2105-11-S1-S49

**Published:** 2010-01-18

**Authors:** MS Vijayabaskar, Saraswathi Vishveshwara

**Affiliations:** 1 Molecular Biophysics Unit, Indian Institute of Science, Bangalore 560012, India

## Abstract

**Background:**

Thermophilic proteins sustain themselves and function at higher temperatures. Despite their structural and functional similarities with their mesophilic homologues, they show enhanced stability. Various comparative studies at genomic, protein sequence and structure levels, and experimental works highlight the different factors and dominant interacting forces contributing to this increased stability.

**Methods:**

In this comparative structure based study, we have used interaction energies between amino acids, to generate structure networks called as Protein Energy Networks (PENs). These PENs are used to compute network, sub-graph, and node specific parameters. These parameters are then compared between the thermophile-mesophile homologues.

**Results:**

The results show an increased number of clusters and low energy cliques in thermophiles as the main contributing factors for their enhanced stability. Further more, we see an increase in the number of hubs in thermophiles. We also observe no community of electrostatic cliques forming in PENs.

**Conclusion:**

In this study we were able to take an energy based network approach, to identify the factors responsible for enhanced stability of thermophiles, by comparative analysis. We were able to point out that the sub-graph parameters are the prominent contributing factors. The thermophiles have a better-packed hydrophobic core. We have also discussed how thermophiles, although increasing stability through higher connectivity retains conformational flexibility, from a cliques and communities perspective.

## Background

Proteins are macromolecules that preserve their structural integrity to perform functions. Thermophilic proteins function at higher temperatures than normal life forms. Although they are structurally, functionally and most instances sequentially homologous to their mesophilic partners, they have optimal catalytic activity above 60°C [1]. The comparative studies of thermophiles and mesophiles, to identify the factors contributing to the stability of the thermophiles, have been carried out at different levels from the genomic sequence level [2], structure level [3] to experimental elucidations [4]. These studies indicate that thermophilic proteins have good hydrophobicity with propensity towards branched side chains, better packing with fewer loops and less cavities, more helical content, increased hydrogen bonding, and high occurrence of charged residues resulting in high electrostatic interactions. Although we have different views on the forces contributing to stability, we do not have a consolidated view of them [3].

Protein Structure Networks (PSNs) have been used extensively to understand the stability in protein structures [5,6]. Protein structure is a resultant of complex intermolecular interactions. PSNs are convenient because this complexity is simplified as edges between nodes. Earlier, PSNs have considered contact based parameters to define edges [5-7].

In this study we have used energies to construct structure networks, known as Protein Energy Networks (PENs), for the first time. Since different types of interactions manifest eventually as interacting energies, we have tried to remove the ambiguities of defining each interaction (VdW, electrostatics, hydrogen bonding) separately, by considering energies calculated using classical force fields to define edges. We were also able to define Lennard-Jones dominant interaction and electrostatics dominated interaction regions in PENs, details of which will be presented elsewhere (work in progress).

In this study we have simulated twelve thermophile-mesophile pairs to obtain their equilibrium ensembles. These ensembles were then used to generate PENs of each protein. Parameters representing the whole protein network such as largest connected component, and parameters focusing on sub-graphs such as clusters, cliques and communities and node specific parameters like hubs are used to obtain structural insights on the stability of thermophilic proteins as compared to their mesophilic homologues.

In this comparative network study, we find that cluster population and clique population, along with community of cliques to be the major factors contributing to the stability of thermophiles. The thermophiles appear to have a highly packed hydrophobic core, by employing amino acid hotspots, thus increasing the enthalpy change between the folded and unfolded states, supporting previous studies [8]. The thermophiles seem to have low energy communities, and segregated high-energy electrostatic cliques, implying that they prefer to maintain a degree of conformation plasticity whilst increasing stability, probably for performing their functions. We have also seen global network connectivity change in some thermophiles, supporting earlier suggestions on global evolution for thermal adaptation [9]. Thermophiles seem to employ more than one of these methods to increase their stability.

## Methods

### Dataset

Twelve protein pairs with similar structure and function, one from thermophilic organism and the other from a mesophile was taken for further analysis. The dataset is derived from an earlier work by Kannan N and Vishveshwara S [6]. The information on this derived dataset is given in Table 1.

**Table 1:** Dataset taken for analysis

Adenylate Kinases 1zip/1ak2	Subtilisin 1thm/1st3	Carboxypeptidases 1obr/2ctc
Neutal Proteases 1thl/1npc	Phosphofructo Kinases 3pfk/2pfk	Lactate Dehydrogenases 1ldn/1ldm

Glyceraldhyde-3-Phosphate Dehydrogenases 1vc2/1gad	3-Phosphoglycerate Kinases 1php/3pgk	Lipomide Dehydrogenases 1ebd/1lvl

Endo-1,4-Beta Xylanases 1yna/1xyn	Triose Phosphate Isomerases 1btm/7tim	Signal Recognition Particle Receptor 1ffh/1fts

The order of thermophile (bold)/mesophile pairs is the same as the order followed in generating all the Additional Figures (Fig S1 to S6).

### Protein Energy Network

The interaction energy is calculated as a summation of short range Lennard-Jones and Coulombic interactions, averaged over an ensemble of structures generated by a constant temperature (300 K), explicit solvent MD simulation performed for 2 nson each protein system, out of which the initial 1 ns was allowed for the system to equilibrate. The equilibrium ensemble from 1 ns to 2 ns was taken for further energy calculations (Comparison of interaction energies of 2 ns simulations with prolonged 10 ns simulations for Adenylate Kinases gave a high correlation of 0.97 (thermophile) and 0.94 (mesophile). This result indicates that the simulation for 2 ns as carried out in this study is sufficient). The simulation and energy calculations were performed using GROMACS [10]. The final graph we obtain is a complete weighted graph of the protein in which the weight of an edge is given by the interaction energy (E_*ij*_) between the amino acids, given in Equation 1.(1)

where, *V*_*LJ*_*(r*_*ij*_) and *V*_*c*_*(r*_*ij*_) are the potential energies due to Lennard-Jones interactions and Coulombic interactions respectively, of residues *i *and *j*, averaged over the ensemble.

Similarly, we have constructed PENs in which we have considered only LJ interactions (*V*_*LJ*_*(r*_*ij*_)) and Coulombic interactions (*V*_*c*_*(r*_*ij*_)) respectively. The details of these graphs are not discussed.

The weighted PEN can further be converted into an unweighted PEN_*e*_, where *e *is the highest energy that can exist between *'i' *and *'j' *to draw an edge between them. For example, PEN_-15_, is a graph where, there exists an unweighted edge between *'i' *and *'j'*, if E_*ij *_≤ -15 KJ/mol. The PEN_*e *_is thus mathematically represented as an adjacency matrix (Equation 2), where(2)

We use the term "low energy" to denote low negative energies (for eg. -5 KJ/mol) and "high energy" for high negative energies (for eg. -25 KJ/mol).

### Clusters

Clusters are the connected components in a PEN_*e *_and can be identified using standard DFS algorithm [11].

### Hubs

Hubs are highly connected nodes in the network. In packing based Protein Structure Network (PSN) studies, a node is declared a hub if its degree is at least 4 [5]. The same definition is being followed here because of similar packing constraints of the proteins analyzed.

### Cliques and communities

Cliques are sub-graphs, having the maximum connectivity among them. For PEN_*e*_, we identify the *k*-cliques using CFinder [12]. For example, for a clique of size *k*, there will be *k *× (*k*-1)/2 edges among the nodes. Two cliques are said to be adjacent if they share *k*-1 nodes. A community is a collection of adjacent *k*-cliques.

### Size

The size of a graph/network or a sub-graph (clusters, cliques and communities) is the total number of nodes in it.

## Results and discussion

**Figure 1:**
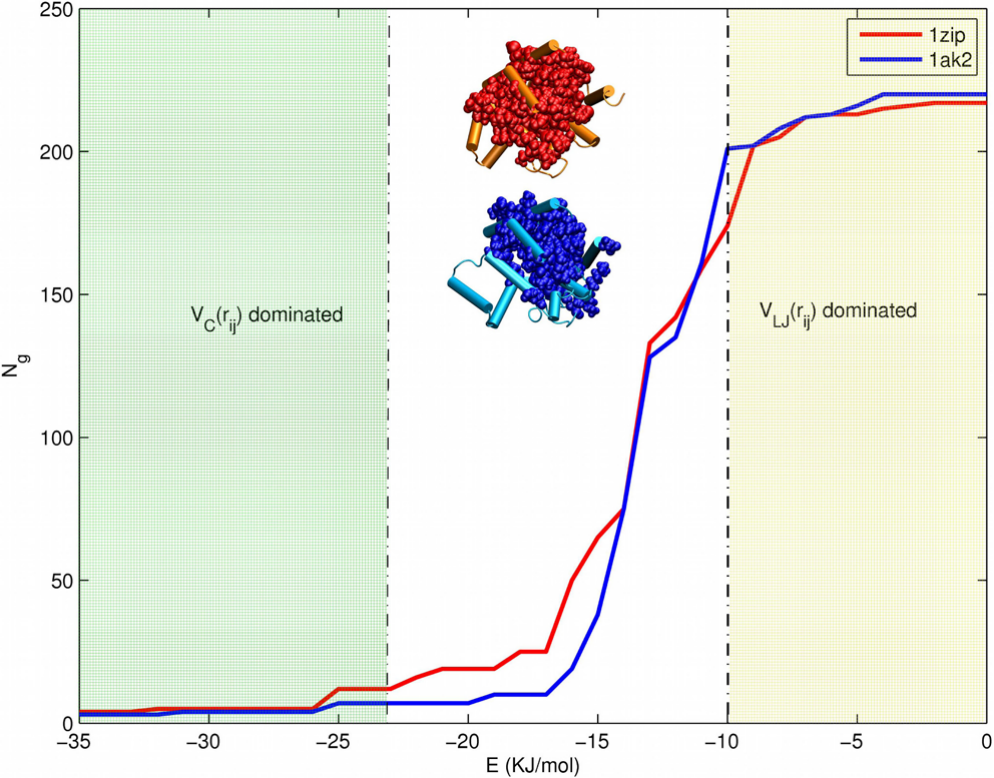
**Transition profile of largest connected component in Adenylate Kinases**. Largest Connected Component (LCC) profile of thermophile(1zip, also orange cartoon) and mesophile(1ak2, also cyan cartoon) Adenylate Kinases is shown in the figure. The thermophile has a larger LCC (shown as red VdW representation over the cartoon representation of the protein) than the mesophile (shown as blue VdW spheres). The LCC represented in VdW spheres are for PEN_-14_. The region below the transition is the low energy region (yellow) where LJ interactions dominate. The high-energy region (green) beyond ~-25 KJ/mol is dominated mainly be salt-bridge interactions. The region of transition is in between these low and high energy regions.

PENs are obtained for the proteins in the dataset as given in Methods section. The interaction energies in PENs mostly range from 0 KJ/mol to -35 KJ/mol, in which the low (negative) energy region (> -10 KJ/mol) is dominated by Van der Waals interaction and the higher (negative) energy region (< -20 KJ/mol) is dominated by electrostatic interactions (Fig 1). The PENs are then analyzed for largest connected component size, largest community size, clusters, cliques and hub population changes, as a function of *'e'*. The results obtained are compared between each thermophile-mesophile pair to obtain insights into their stability differences, from a network perspective. A comparison of different parameters for PEN_*e *_of each pair is given in Additional file 1, Table S1.

### Largest connected component (LCC) transition profile

The Largest Connected Component is a very important parameter in network analysis since it provides information on the connectivity of the network [5,11]. The Largest Connected Component (LCC) is obtained as a function of *'e'*. The LCC is well connected at low energy regions but breaks up at the transition region (Fig 1). The LCC transition profile comparison shows that thermophiles of Adenylate Kinases (Ad Kinases) (Fig 1), Subtilisins, Carboxypeptidases, PhosphoFructo (PF) Kinases and Endo-1,4-Beta Xylanases (E14B Xylanases) show a more connected LCC than mesophiles (Fig S1). From LCC profile we observe that global evolution from mesophile to thermophile is not a prominent contributing factor, nonetheless we observe certain thermophiles following this behavior (Fig 1 and Additional file 1, Fig S1).

### Cluster population

**Figure 2:**
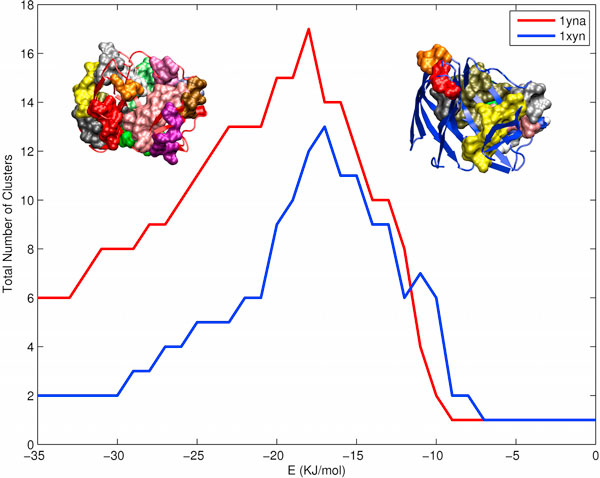
**Cluster population change with 'e' in Endo-1,4-Beta Xylanases**. Cluster population change as a function of *'e' *denoted in KJ/mol in E14B Xylanases is shown as a line plot. The graph shows the increased number of clusters in thermophile(1yna, also red cartoon) as compared to mesophile(1xyn, also blue cartoon) protein. The isolated clusters in each protein (for PEN_-20_) are represented as Surf in various colors for both 1yna (red cartoon) and 1xyn (blue cartoon) to highlight the increased occurrence of clusters in the thermophilic E14B Xylanase.

Clusters are autonomously connected units in PENs. They provide us information on what amino acids are interconnected to stabilize which part of the protein structure [6]. The more clusters there are in a PEN, the more segregated the stability of the protein is. Clusters for a PEN_*e *_are obtained using Depth First Search as given in Methods Section and the total number of clusters (only if cluster size is atleast 3) is calculated as a function of *'e' *(Fig 2, clusters segregate at high *'e'*). The cluster population profiles show a consistent pattern of thermophiles having higher number of clusters than mesophiles (except Ad Kinases and Carboxypeptidases) (Additional file 1, Fig S2). The profiles mostly show that (i) the population of clusters peaks higher for thermophiles than mesophiles (seen in E14B Xylanases, Li Dehydrogenases), (ii) the thermophile cluster population peak is shifted towards higher energy (seen in Lactate Dehydrogenases (L Dehydrogenases), 3 PhosphoGlycerate Kinases (3PG Kinases)) or (iii) the number of clusters at higher energies (< -20 KJ/mol) are more than that of its mesophile counterpart (Additional file 1, Fig S2). Fig 2 shows an example of the cluster population profile of E14B Xylanases where it follows all the three patterns mentioned above. The increased population of electrostatics dominated clusters at high energy levels (< -20 KJ/mol), like in 3PG Kinases, can highly stabilize the thermophile protein. In general the higher cluster population in thermophiles suggests enhanced stability due to increased number of segregated groups of interactions.

### Largest community transition profile

**Figure 3:**
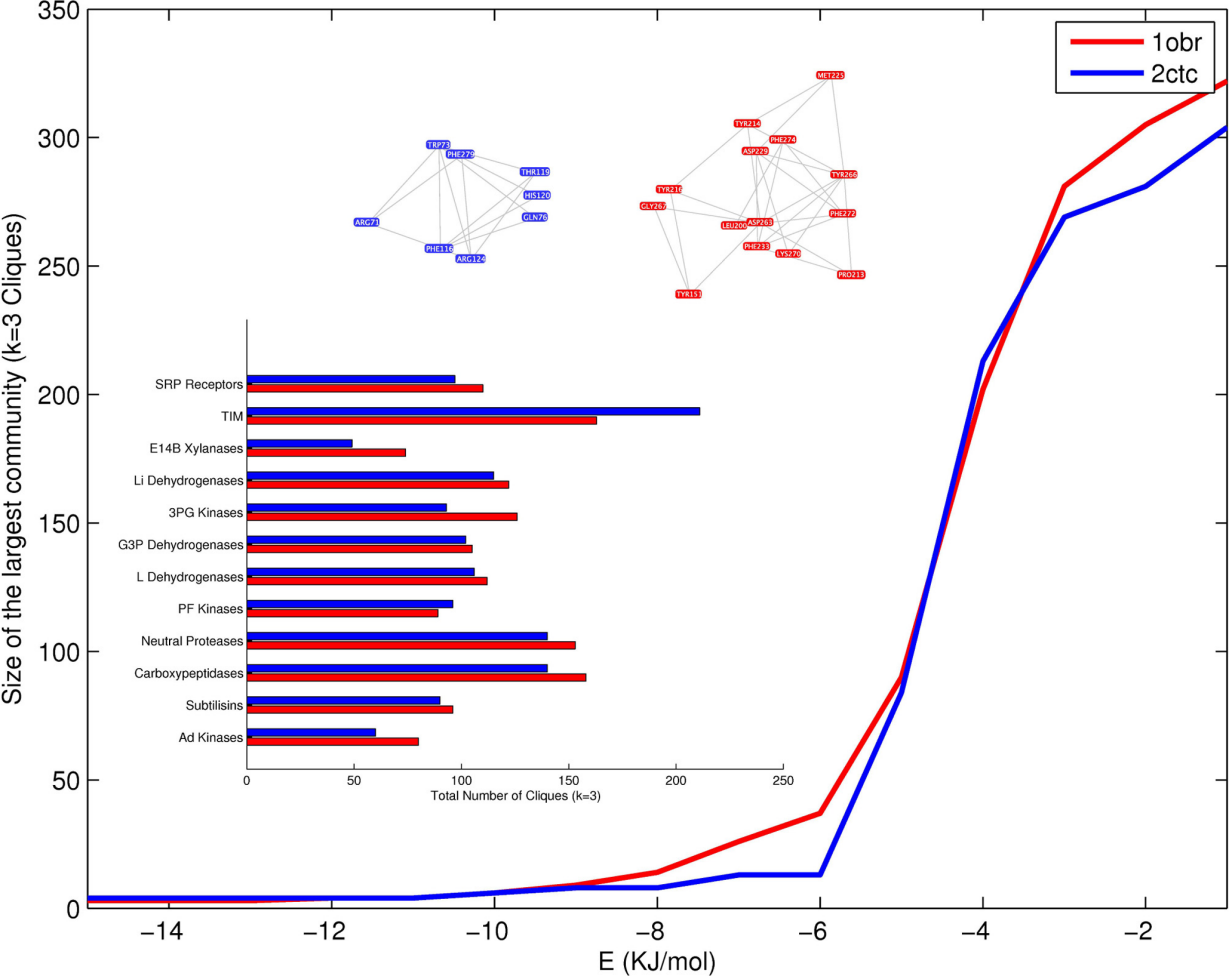
**Largest community transition profile in Carboxypeptidases**. The size of the largest community (of *k *= 3 cliques) for Carboxypeptidases, is plotted as a function of *'e'*. The largest community (*k *= 3 cliques for PEN_-8_) for thermophile, 1obr (red nodes), is almost always bigger than the mesophile, 2ctc (blue nodes). The bar diagram given below is the total number of cliques for PEN_-6_. This shows the increased number of cliques in thermophilic proteins (except TIM and PF Kinases) at low energies. This increased clique population is clearer from Fig S5.

The cliques are rigid sub-graphs in a network [13]. And hence, a community is a collective rigid sub-graph of PEN_*e*_, thus giving stability to the protein. The communities in a PEN_*e *_are identified as given in Methods Section. The size of the largest community (of *k *= 3 cliques) is plotted as a function of *'e'*, where the community size is large at lower energies and breaks down as we decrease *'e' *(Fig 3). From, the community transition profile comparison of thermophiles and mesophiles, we find that the thermophiles perform similar or better than the mesophiles, having larger community sizes in PENs at lower energy levels. For example, the thermophilic Carboxypeptidase has a larger community (*k *= 3 cliques) at a lower energy regime (*e *= 0 to -8 KJ/mol) than the mesophile pair (Fig 3). An additional mesophilic carboxypeptidase (PDBID: 2piz) with high structural homology but a sequence homology of only 46% shows the same profile as the mesophile showing that the mesophiles indeed follow the same trend which is distinct to that of their thermophilic homolog (Additional file 1, Figure S3). There are some exceptions like L Dehydrogenases and Signal Recognition Particle Receptors (SRP Receptors) (Additional file 1, Fig S4). The presence of communities only in the low energy region, suggests absence of electrostatic influence and hence the non-preference to electrostatic interactions aggregating to form very rigid networks.

### Clique population

Although, communities provides insight into how rigid sub-graphs collate to provide rigidity to the protein, isolated cliques can also provide similar rigidity to parts of the protein, empowering islands in protein structures to withstand extreme temperatures. From profiling the population change of cliques with changes in *'e'*, we were able to capture this effect in proteins. Analysis of the clique population profile in PEN_*e *_shows that almost all thermophiles with the exception of TIM, shows increased population of cliques at low energies (Additional file 1, Fig S5 and Fig 3). Unlike community transition profile, we see considerable number of cliques dominated by electrostatics (by constructing PENs with E_ij _= *V*_*C*_*(r*_*ij*_)). Conformational plasticity might be a possible factor influencing this behavior. The thermophilic proteins seem to employ low energy cliques and communities to maintain stability, but use only segregated high energy electrostatics dominated cliques to maintain rigidity. This strategy might help thermophiles to maintain stability but retain a degree of flexibility, enabling them to function.

### Hub population

**Figure 4:**
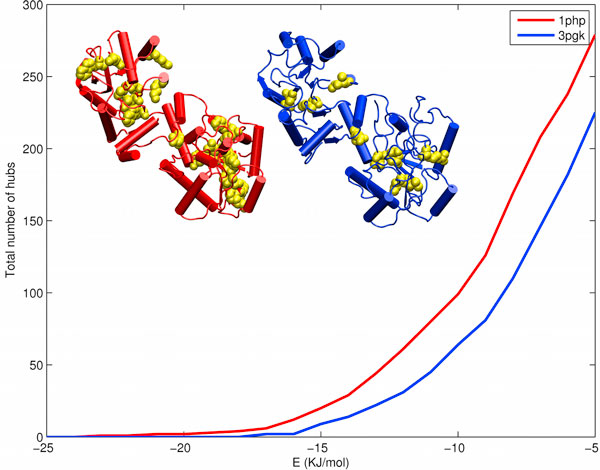
**Hub population change as a function of 'e' in Phosphoglycerate Kinases**. The hub population change as a function of *'e' *for Phosphoglycerate Kinases is shown in the line plot. The hubs in PEN_-15 _(yellow VdW representation) in thermophilic PG Kinase, 1php (red cartoon representation), is considerably higher than in the corresponding mesophile, 3pgk (blue cartoon representation).

Hubs are node specific property in a network. In real world networks, hubs are considered to provide resilience to the networks against random attacks [14], in the case of proteins against random mutations [5]. In a PEN_*e*_, it can represent the structurally and possibly functionally important residues in the protein. The number of hubs plotted as a function of *'e' *can give us information on the amount of structural resilience a protein (PEN) can have against external perturbations. Thermophiles show higher hub population than mesophiles (with exceptions like 3PF Kinases, Glyceraldhyde-3-Phosphate Dehydrogenases (G3P Dehydrogenases) (Additonal file 1, Fig S6)). Fig 4 shows that the hub population of thermophilic 3PG Kinase is more than the mesophilic counterpart, both at the low energy and at the transition regions. An interesting observation is that most of the thermophiles have consistently higher number of hubs than mesophiles in lower energy region if we remove electrostatics from PEN calculation (i.e. E_ij _= *V*_*LJ*_*(r*_*ij*_)), supporting packing based PSN studies by Brinda KV and Vishveshwara S [5]. The above observation suggests that the thermophiles may have more efficiently packed hydrophobic cores than mesophiles, supporting previous studies [2,8,15].

## Conclusion

In this study, we have used Protein Energy Networks for representing interactions in protein structures, to compare a dataset of thermophile-mesophile homologues. Network parameters like the largest connected component, sub-graph properties like clusters, cliques and communities and node specific parameters like hubs were compared across a range of interaction energies, to identify the factors contributing to the enhanced stability of thermophiles. In this work, we have consolidated all the interaction types by using their resultant interaction energies (calculated using classical force fields). This effort has eliminated ambiguities in defining and analyzing interactions separately. Also, we have considered the complexity of amino acid interactions by representing them as networks.

From the results obtained for PENs, we were able see that thermophilic proteins like Adenylate Kinases, Subtilisins, Carboxypeptidases, 3PG Kinases and Endo-1,4-Beta Xylanases show enhanced global connectivity, as seen from their increased LCC transition profiles. But thermophiles seem to prefer more than one factor for stabilization. For example, Adenylate Kinases thermophile seems to employ large communities and increased clique population than the mesophiles, whereas E14B Xylanase thermophile has a larger number cliques and hubs to enhance their stability. The LCC for thermophilic Neutral Proteases and Lipodimide Dehydrogenases are less connected than the corresponding mesophiles. Hence, the notion that evolution may prefer a global change from a thermophile to a mesophile might be more case specific. This was further strengthened by our observations on increased cluster population in thermophiles, showing that they use more autonomous stabilizing units to enhance stability. Also, increased electrostatic clusters in certain thermophiles, at higher energy ranges (< -20 KJ/mol), suggest that they might play a vital role in imparting stability to them, supporting earlier works in this area [16,17].

Thermophilic proteins show a higher clique population than their mesophile homologues. But the clique population and largest community formation are at the low energy regime. PENs have electrostatic cliques but they do not form community. These observations suggest that the thermophilic proteins may employ a higher number of low energy communities to gain increased stability, but refrain from introducing much rigidity to the protein by keeping the high energy electrostatic cliques isolated. This property might allow thermophiles to be more stable but retain flexibility, probably to perform its function.

The studies on hub population suggest that the hubs (especially LJ dominated hubs) in thermophilic proteins are higher than that of mesophiles. Hence, the hydrophobic core connectivity (packing) in thermophilic proteins is better than mesophile partners, probably enabling them to stay folded under harsh conditions. This observation supports many studies on improved core packing in thermophilic proteins [8].

This comparative network based study suggests that global evolution of local enhancements has resulted in an increase in overall network connectivity and hence an increase in global stability of thermophiles. Increase in clusters and hubs in thermophiles bring about these local enhancements. Apart from these changes, thermophiles seem to employ low energy communities (highly connected sub-graphs) to maintain a level of rigidity to the network. Presence of electrostatic clusters, and cliques but absence of communities, shows localized electrostatic interactions, rather than global network influences. And thermophilic proteins seem to have evolved by exploiting more than one of the above-mentioned methods.

## Competing interests

The authors declare that they have no competing interests.

## Authors' contributions

MSV and SV have designed the experiments, analyzed the data and prepared the manuscripts.

## Supplementary Material

Additional file 1 **Network parameter comparison of PENs in thermophilic and mesophilic proteins**. Figure S1 shows the comparison of the largest connected component as a function of 'e' between the thermophile/mesophile homologs. Similarly, Figure S2, S4, S5 and S6 show such comparison for the cluster population, largest community size, clique population and hub population respectively. Figure S3 shows the comparison of different network parameters between one thermophilic and two mesophilic carboxypeptidases. Table S1 gives an overview of the different network parameters (at a specific *'e'*) across the whole dataset, along with some commonly analyzed pairwise interactions. Click here for file
