# Supplementary material for: Comparative analysis of thermophilic and mesophilic proteins using Protein Energy Networks
Source: BMC Bioinformatics. 2010 Jan 18;11(Suppl 1):S49. doi: 10.1186/1471-2105-11-S1-S49 (PMC3009521; doi:10.1186/1471-2105-11-S1-S49)
Supplement: Additional file 1 — Network parameter comparison of PENs in thermophilic and mesophilic proteins. Figure S1 shows the comparison of the largest connected component as a function of 'e' between the thermophile/mesophile homologs. Similarly, Figure S2, S4, S5 and S6 show such comparison for the cluster population, largest community size, clique population and hub population respectively. Figure S3 shows the comparison of different network parameters between one thermophilic and two mesophilic carboxypeptidases. Table S1 gives an overview of the different network parameters (at a specific 'e') across the whole dataset, along with some commonly analyzed pairwise interactions. [file 1471-2105-11-S1-S49-S1.pdf]

Additional file 1 – Network parameter comparison of PENs in thermophilic and mesophilic proteins

Figure S1 - Largest Connected Component transition profile in PEN

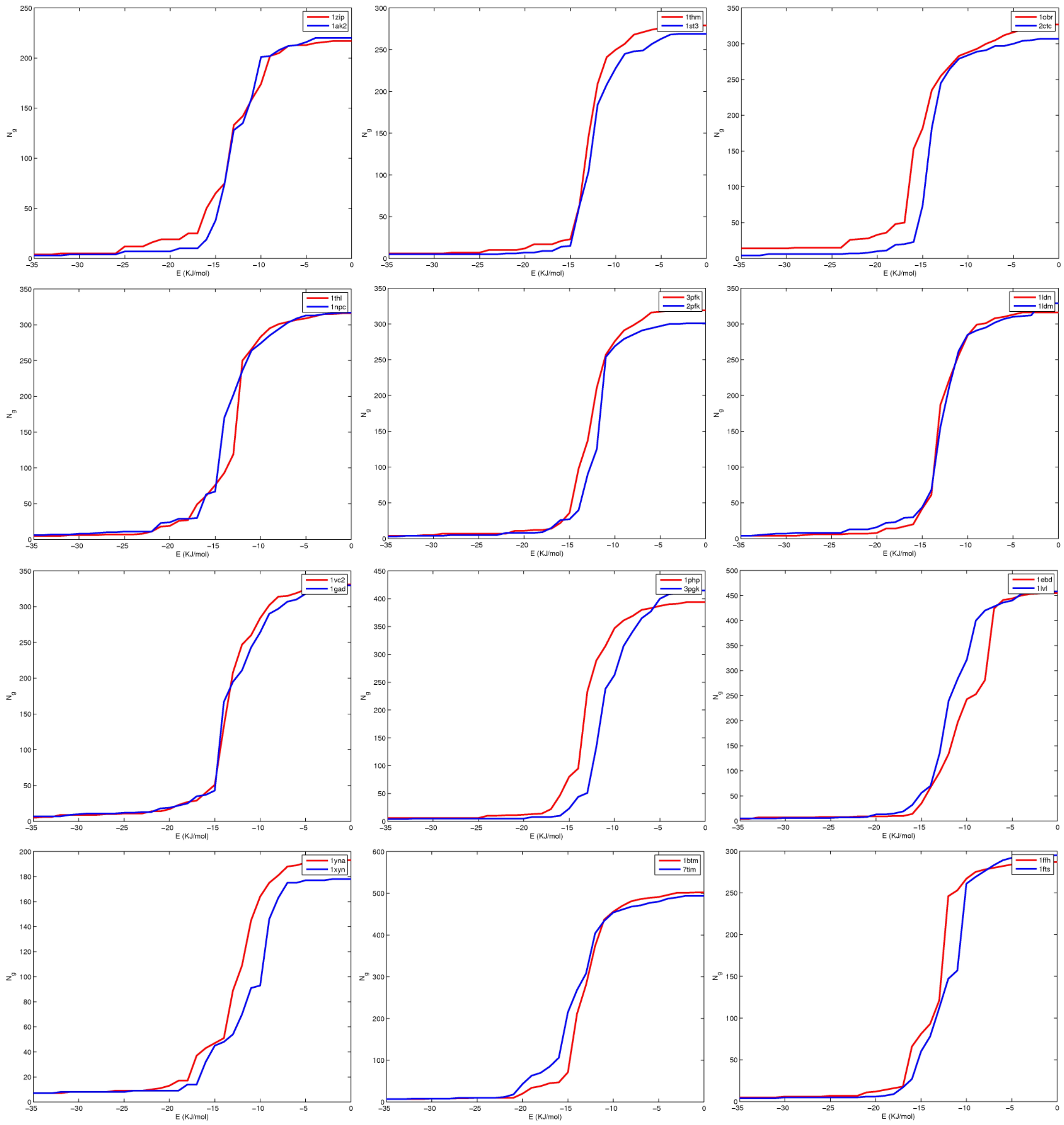

The figure above shows the largest connected component transition as a function of ‘e’ in PENe. ‘e’ is varied from 0 to -35 KJ/mol to cover almost all the interaction range. The figure shows the LCC transition plots for all the twelve thermophile (red solid line)-mesophile (blue solid line) pairs. The figure legends follow the same order from top left to right bottom as given in Table 1.

Figure S2 - Cluster Population Change with 'e' in PEN

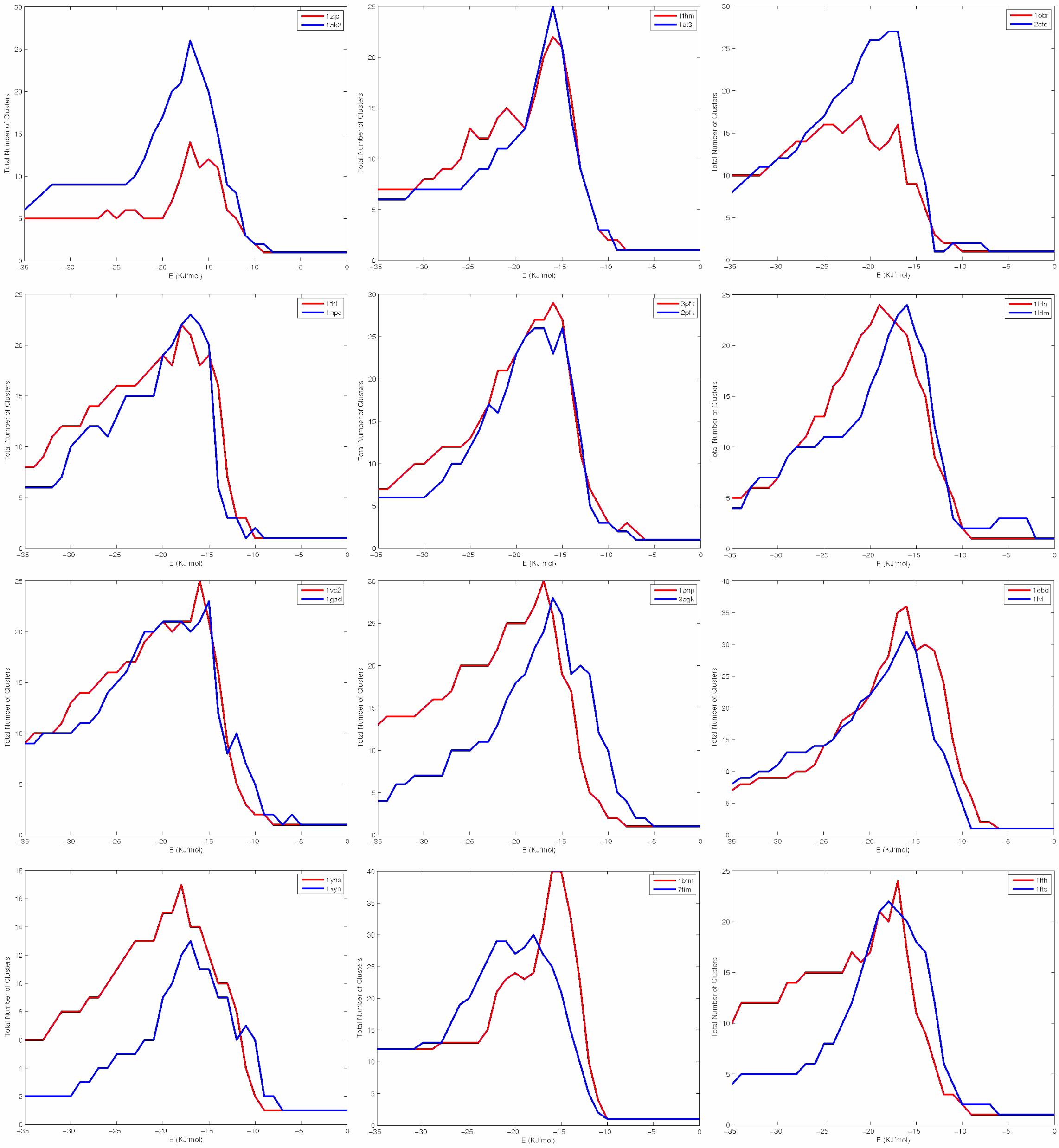

The figure shows the cluster population change as a function ‘e’, in their corresponding PENe. The figure shows the cluster population change plots for all the twelve thermophile (red solid line)-mesophile (blue solid line) pairs. The figure legends follow the same order from top left to right bottom as given in Table 1.

Figure S3 - Comparison of thermophilic carboxypeptidase with two of its mesophilic homologs

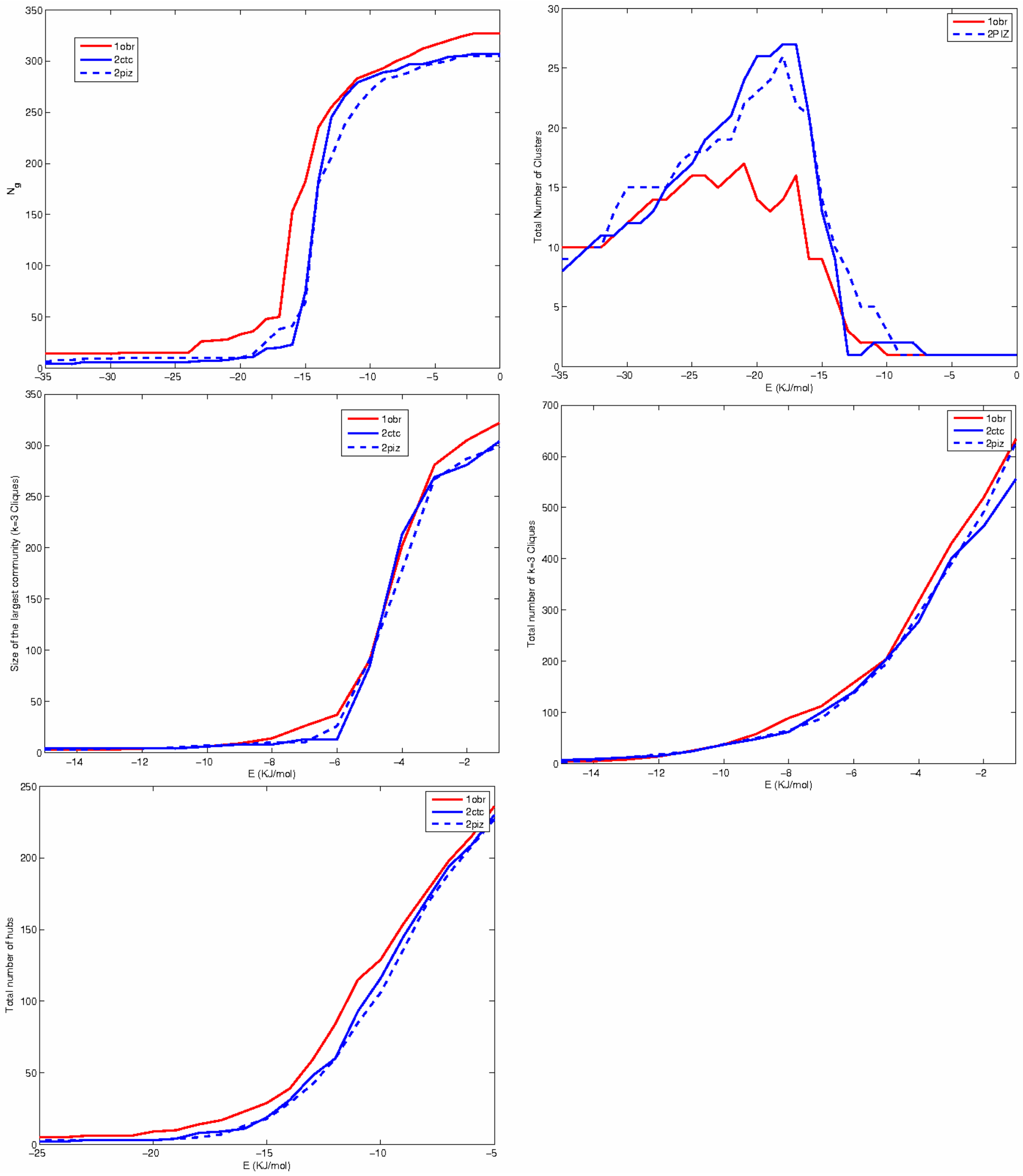

The figures show the comparison of different network parameters between the carboxypeptidases from the thermophile (PDB ID: 1obr, red solid line), with two of its

mesophilic homologs (PDBIDs: 2ctc and 2piz, blue solid line and blue dashed line respectively). It shows the similarity in the behavior of the mesophiles and they are distinct from that of the thermophile. The sequence identities between the thermophile and the mesophiles are (1obr-2ctc = 26.6% and 1obr-2piz = 29%) and the mesophiles are (2ctc-2piz = 45.6%).

Figure S4 - Largest Community transition profile in PEN

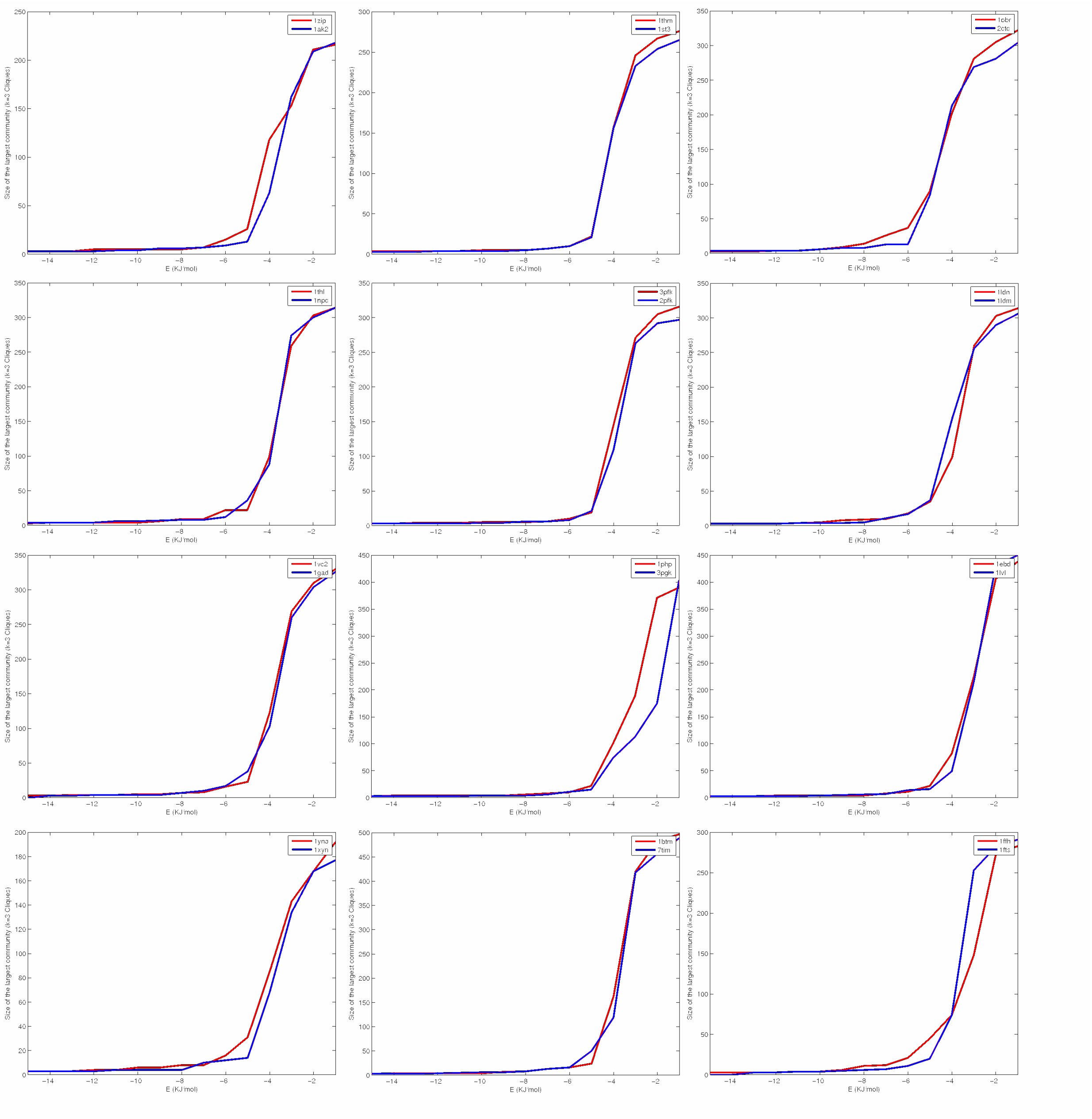

The figure shows the largest community (of k=3 cliques) transition profile as a function of ‘e’ in PENs for each thermophile (red solid line) – mesophile (blue solid line) pair. The figure legends follow the same order from top left to right bottom as given in Table 1.

Figure S5 - Clique Population Change with 'e' in PEN

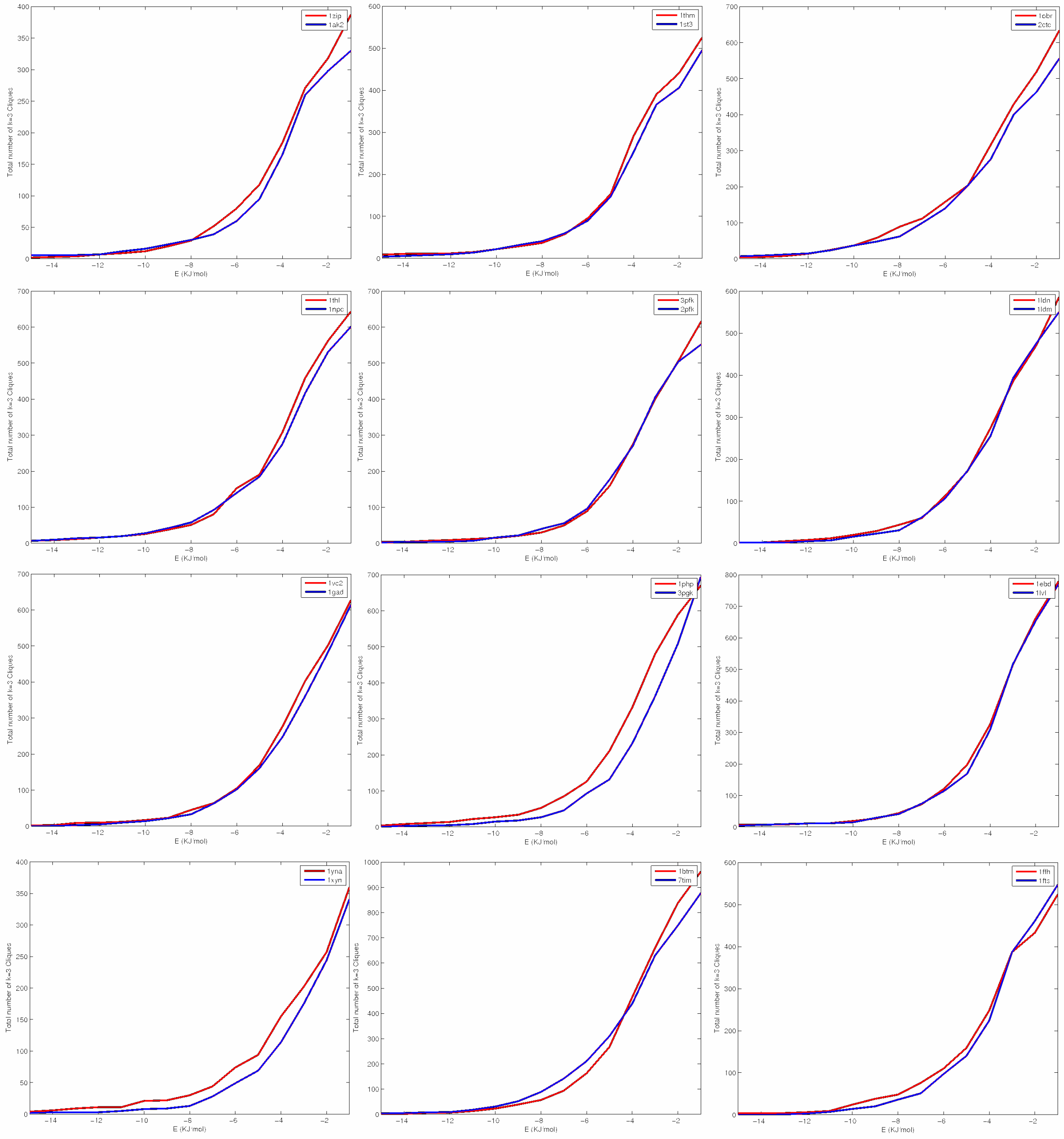

The figure shows the k=3 clique population change with ‘e’ in PENs for all the thermophile (red solid lines) – mesophile (blue solid lines). The figure legends follow the same order from top left to right bottom as given in Table 1.

Figure S6 - Hub Population Change with 'e' in PEN

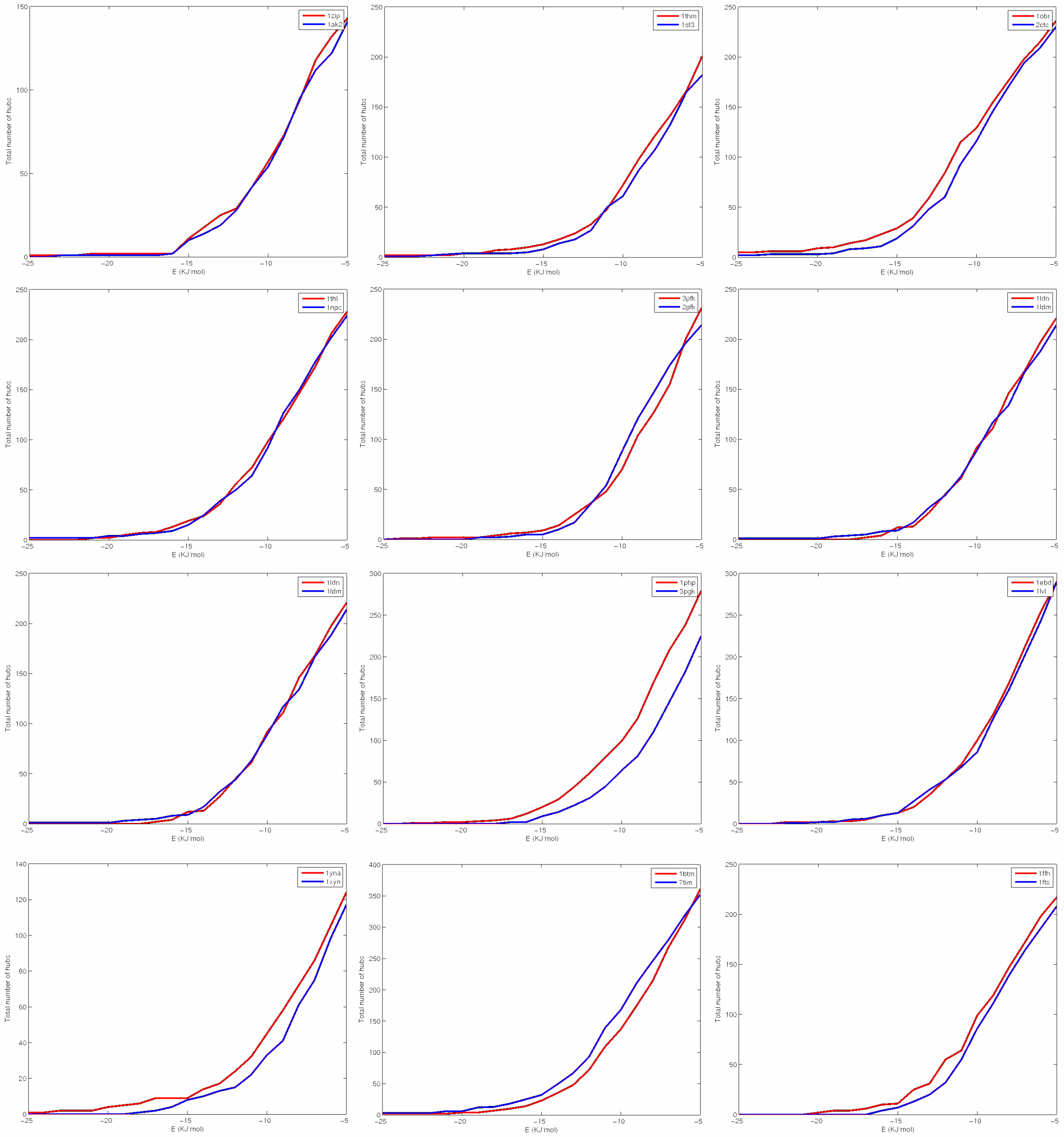

The figure shows the hub population change as a function of ‘e’ for the thermophile (red solid lines) – mesophile (blue solid lines) PENs. The figure legends follow the same order from top left to right bottom as given in Table 1.

Table S1 - Comparison of different network parameters and pairwise interactions between the thermophile mesophile homologs.

Proteins (Thermophile/Mesophile)	Network Parameters Analysis					Pairwise Interaction Analysis		
	Largest Cluster (*e* = -15 KJ/mol)	Total Number of Clusters (*e* = -17 KJ/mol)	Largest Community (*e* = -5 KJ/mol)	Total Number of Cliques (*e* = -5 KJ/mol)	Hubs (*e* = -12 KJ/mol)	[a]Saltbridges	[b]Hydrogen bonds	[c]Disulphide bonds
1zip\1ak2	**65\38**	14\26	**118/95**	**26/13**	**29\28**	12/12	**67/60**	0/1
1thm\1st3	**23\15**	20\21	**153/147**	**22/21**	**33\27**	**9/6**	**115/102**	0/0
1obr\2ctc	**182\74**	16\27	204/204	**90/84**	**84\60**	**17/10**	**140/126**	**2/1**
1thl\1npc	**76\67**	**21\23**	**191/185**	22/36	**56\50**	13/15	121/127	0/0
3pfk\2pfk	**36\27**	**27\26**	160/178	19/21	**36\35**	**10/8**	89/92	0/0
1ldn\1ldm	42\44	22\23	172/173	35/37	**45\44**	**12/7**	71/80	0/0
1vc2\1gad	**51\43**	**21\20**	**169/161**	23/38	**53\34**	11/16	120/124	0/0
1php\3pgk	**80\23**	**30\24**	**211/132**	**22/15**	**61\31**	**19/2**	**118/30**	0/0
1ebd\1lvl	35\56	**35\29**	**198/169**	**22/16**	53\53	**14/10**	**102/98**	0/1
1yna\1xyn	**47\45**	**14\13**	**94/69**	**31/14**	**24\15**	**4/1**	**81/49**	**1/0**
1btm\7tim	71\215	**31\27**	267/310	24/50	72\93	11/13	69/81	0/0
1ffh\1fts	**81\60**	**24\21**	**159/140**	**45/20**	**55\32**	**25/7**	**104/47**	0/0

The bold observations indicate that the thermophile dominate the mesophilic homolog in the corresponding parameter at a specific *'e'*.

[a] The salt bridge interactions are calculated using VMD with O-N distance <= 3.2 Å

[b] The Hydrogen bonds are calculated using Hbplus v3.15 with D-A distance <= 3.9 Å and DHA angle >= 120°

[c] The disulphide bond information was obtained from the PDB file descriptions
